# Evaluation of the relationship between oral health-related quality of life (OHRQoL) and skeletal malocclusion – a pilot study

**DOI:** 10.1080/07853890.2021.1897346

**Published:** 2021-09-28

**Authors:** Dinis Pereira, Vanessa Machado, João Botelho, Luís Proença, Ana Delgado, José João Mendes

**Affiliations:** a Egas Moniz Dental Clinic (EMDC), Egas Moniz Cooperativa de Ensino Superior, Caparica, Portugal; b Centro de Investigação Interdisciplinar Egas Moniz (CiiEM), Egas Moniz Cooperativa de Ensino Superior, Caparica, Portugal

## Abstract

**Introduction:**

The impact of dental malocclusion in the oral health-related quality of life (OHRQoL) has been described in the literature [1,2]. However, the impact of severity of skeletal malocclusion on the OHRQoL has never been addressed. Therefore, this exploratory study aimed to evaluate the relationship between patient perception of OHRQoL and skeletal malocclusion.

**Materials and methods:**

This study was approved by the Egas Moniz Ethics Committee. This cross-sectional observational study involved patients that sought orthodontic treatment between January and April 2019, at the Orthodontic Care Consultation of Egas Moniz Dental Clinic (Monte de Caparica – Almada, Portugal). Exclusion criteria were patients with severe diseases, craniofacial abnormalities, cognitive deficits, caries, periodontal diseases, and previous orthodontic treatment history. A total of 19 patients met the criteria and were enrolled in the study. OHRQoL was assessed through the Oral Health Impact Profile – Portuguese validated version (OHIP-14) [3] and skeletal malocclusion by cephalometric Steiner criteria [4]. Resulting data were submitted to descriptive and inferential statistical analysis.

**Results:**

The sample included 7 (37%) males and 12 (63%) females, with a mean age of 27.9 years. Patient distribution among Steiner’s classes was as follows: 10 (52.6%) Class I, 7 (36.8%) Class II and 2 (10.6%) in Class III. The OHIP-14 total score ranged from 0 to 49. Significant differences were found for the OHRQoL domain related to psychological discomfort, among the three skeletal classes (*p* = .027), with lower median scores obtained for Class II (0) when comparing to Class I (3.5) and Class III (4.5). All other OHRQoL domains were not found to be significantly different among the skeletal classes (*p* > .05).

**Discussion and conclusions:**

For the first time, OHRQoL’s psychological discomfort domain was found to be significantly different when considering the severity of skeletal malocclusion. Overall, Class II patients have less psychological discomfort with their clinical condition than the remaining malocclusion patients. This is the first study to evaluate the OHRQoL quantifying the severity of skeletal malocclusion. This pilot study will serve as the basis for larger-scale studies in order to comprehensively validate these preliminary findings.
